# Extracellular circular RNA profiles in plasma and urine of healthy, male college athletes

**DOI:** 10.1038/s41597-021-01056-w

**Published:** 2021-10-28

**Authors:** Elizabeth Hutchins, Rebecca Reiman, Joseph Winarta, Taylor Beecroft, Ryan Richholt, Matt De Both, Khalouk Shahbander, Elizabeth Carlson, Alex Janss, Ashley Siniard, Chris Balak, Ryan Bruhns, Timothy G. Whitsett, Roger McCoy, Matthew Anastasi, April Allen, Brian Churas, Matthew Huentelman, Kendall Van Keuren-Jensen

**Affiliations:** 1 Neurogenomics Division, TGen, 445 N. 5th St., Phoenix, AZ 85004 USA; 2 Arizona State University Sports Medicine, 323 E Veterans Way, Tempe, AZ 85281 USA

**Keywords:** Genetics research, Transcriptomics

## Abstract

Circular RNA (circRNA) are a recently discovered class of RNA characterized by a covalently-bonded back-splice junction. As circRNAs are inherently more stable than other RNA species, they may be detected extracellularly in peripheral biofluids and provide novel biomarkers. While circRNA have been identified previously in peripheral biofluids, there are few datasets for circRNA junctions from healthy controls. We collected 134 plasma and 114 urine samples from 54 healthy, male college athlete volunteers, and used RNASeq to determine circRNA content. The intersection of six bioinformatic tools identified 965 high-confidence, characteristic circRNA junctions in plasma and 72 in urine. Highly-expressed circRNA junctions were validated by qRT-PCR. Longitudinal samples were collected from a subset, demonstrating circRNA expression was stable over time. Lastly, the ratio of circular to linear transcripts was higher in plasma than urine. This study provides a valuable resource for characterization of circRNA in plasma and urine from healthy volunteers, one that can be developed and reassessed as researchers probe the circRNA contents of biofluids across physiological changes and disease states.

## Abstract (summary)

**Table:** 

Measurement(s)	transcriptome • RNA(circular)
Technology Type(s)	RNA sequencing
Factor Type(s)	biofluid
Sample Characteristic - Organism	Homo sapiens

Machine-accessible metadata file describing the reported data: 10.6084/m9.figshare.14991822

## Background and Summary

The advent of next-generation sequencing has spurred the discovery of a growing list of RNA biotypes, many of which are detectable across species, detected in numerous biofluids, and have biological function. While many studies have focused on microRNAs (miRNA), several other small RNA species (e.g. piwi-interacting RNAs (piRNA), tRNA fragments, and Y RNA fragments have been detected across a range of biofluids and are being developed as clinical biomarkers^1–4^. In addition to these linear RNAs, the discovery and detection of circular RNAs (circRNA), those with a covalently closed loop structure, have gained attention.

CircRNAs were initially discovered by electron microscopy, in the 1970s, as viroid molecules^5^. Nearly two decades later, circRNA were identified for a handful of mammalian genes^6–8^. Though initially thought to be rare splicing events, circRNAs have recently been identified as an abundant, endogenous RNA species in a number of organisms from Archaea to yeast, plants, worms, flies, fish, and mammals^9–11^. Additionally, circRNAs are abundantly expressed in a number of human tissues and cell types, and circRNA expression changes during development, and as a response to extrinsic factors such as stress, immune response, and hormonal stimuli^12–17^. These endogenous RNAs are characterized by their circular structures, which are formed by a back-splicing event that covalently links the 3′ “tail” splice donor with the upstream 5′ splice acceptor “head” of the transcript, forming a back-spliced, or “head-to-tail” junction. While circRNA function is still being elucidated, there are examples of circRNA inhibiting microRNA, regulating alternative splicing, and modulating the expression of parental genes^18–22^.

In comparison to their linear counterparts, circRNA transcripts can be more abundant and have greater stability as they are resistant to linear decay mechanisms and do not contain 5′-3′ polarity nor polyadenylated tails^14,21,23,24^, suggesting feasibility as stable biomarkers. CircRNA stability and detection in biofluids, saliva^25^, blood^24,26–30^, and urine^31–33^, comes, in part, from their being protected in extracellular vesicles^28,34–36^. Changes in circRNA expression is altered in multiple diseases, including preeclampsia, glioblastoma and colorectal cancer^30,37,38^. More recently, circRNAs in tumor tissues, as determined by next-generation sequencing, correlated with disease progression^39,40^. Urine circRNAs correlated with kidney rejection post-transplant^31^, while differentially expressed circRNAs have been determined in plasma exosomes of lung cancer patients versus controls^41^. Most studies of circRNA have small sample sizes or are based on targeted microarray data, rather than discovery-based methods. This dataset includes more than 100 samples from 54 volunteers from two easily accessible biofluids (plasma and urine). In some cases, multiple samples were collected from the same participant longitudinally, allowing us to assess the reliability of circRNA detection in biofluids.

**Fig. 1:**
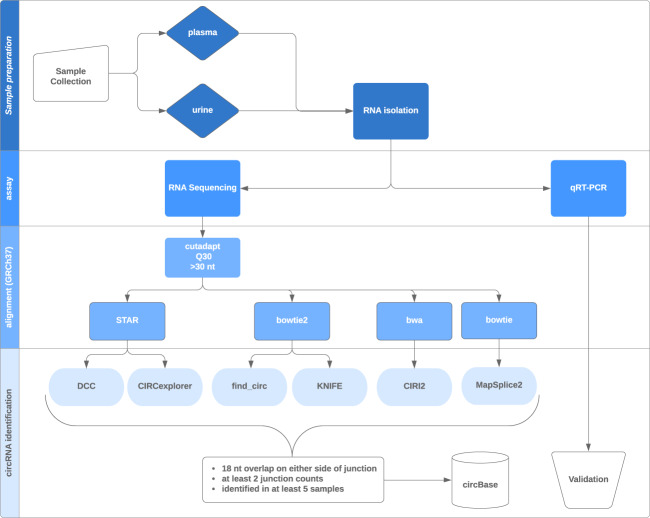
Study Workflow.

**Fig. 2:**
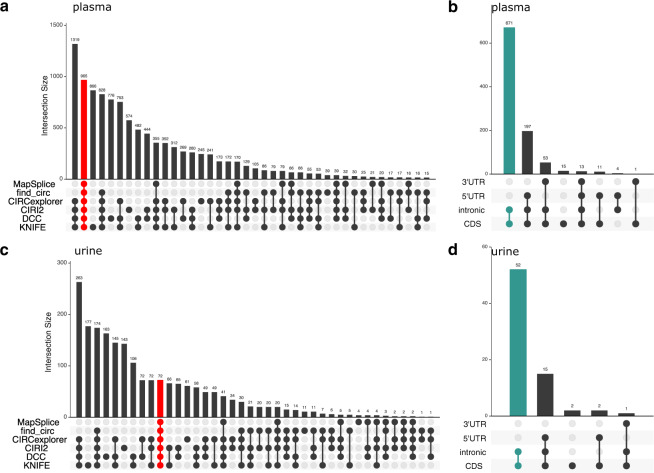
CircRNAs were predicted from 134 plasma (**a**,**b**) and 114 urine (**c**,**d**) samples using 6 different bioinformatic tools. 965 circRNA were identified by all 6 tools in plasma (a; red bar), and 72 circRNA were identified by all 6 tools in urine (**c**; red bar). Genomic features located within predicted back-spliced junctions in plasma (**b**) and urine (**d**), respectively.

The stability and abundance of circRNAs led us to investigate detection in two easily accessed biofluids: plasma and urine. As the volunteers were part of a larger study elucidating concussion biomarkers in male, college athletes, the samples are derived from young (18–25), healthy, male volunteers as depicted in Table 1. The longitudinal sample collections of plasma and urine are depicted in Online-only Table 1, including the number of circRNAs identified in each biofluid and those circRNAs observed concurrently in the biofluids. We identified circRNA in plasma (n = 134) and urine (n = 114), using RNAseq data followed by one of six different bioinformatic tools (Fig. 1). The intersection of the 6 bioinformatic tools provides a catalog for circRNA in plasma (Fig. 2a) and urine (Fig. 2c).

**Table 1:** Healthy Participant Characteristics.

Participant	# plasma samples	# urine samples	Age	Racial or Ethnic Category	Protocol	dbGaP Participant ID
001	1	1	19	AA	RNAseq	2048063
002	1	1	20	AA	RNAseq	2048064
004	3	3	22	AA	RNAseq	2048065
005	5	1	21	AA	RNAseq	2048066
006	3	3	22	W	RNAseq	2048067
007	3	5	20	AA	RNAseq	2048068
008	2	0	21	H	RNAseq	2048069
010	3	2	21	AA	RNAseq	2048070
011	4	6	20	AA	RNAseq	2048071
013	2	5	20	HAW	RNAseq	2048072
014	3	4	22	W	RNAseq	2048073
015	1	1	20	HAW	RNAseq	2048074
019	3	1	23	AA	RNAseq	2048075
022	2	6	N/A	W	RNAseq	2048076
023	0	1	19	AA	RNAseq	2048077
024	7	6	21	AA	RNAseq	2048078
025	2	2	20	AA	RNAseq	2048079
029	2	2	21	W	RNAseq	2048080
030	3	2	22	AA	RNAseq	2048081
031	5	2	23	W	RNAseq	2048082
036	0	1	21	W	RNAseq	2048083
039	4	3	22	W	RNAseq	2048084
042	1	0	23	AA	RNAseq	2048085
044	1	2	22	W	RNAseq	2048086
045	5	5	20	W	RNAseq	2048087
046	7	4	22	AA	RNAseq	2048088
048	2	1	21	AA and H	RNAseq	2048089
049	5	3	22	AA	RNAseq	2048090
170	1	1	18	Asian	RNAseq	2048091
201	3	5	N/A	AA	RNAseq	2048092
202	0	3	22	AA	RNAseq	2048093
203	9	4	22	AA	RNAseq	2048094
204	1	0	N/A	AA	RNAseq	2048095
205	1	1	N/A	N/A	RNAseq	2048095
206	5	0	20	AA	RNAseq	2048097
207	2	1	20	HAW	RNAseq	2048098
208	1	0	19	AA	RNAseq	2048099
209	1	1	20	W and H	RNAseq	2048100
210	1	1	21	AA	RNAseq	2048101
211	1	0	N/A	N/A	RNAseq	2048102
212	1	0	20	W	RNAseq	2048103
213	1	3	22	AA	RNAseq	2048104
214	0	2	20	W	RNAseq	2048105
215	0	1	20	H	RNAseq	2048106
216	1	3	19	AA	RNAseq	2048107
218	1	1	20	AA	RNAseq	2048109
220	1	0	N/A	N/A	RNAseq	2048111
221	8	3	19	AA	RNAseq	2048112
222	1	0	N/A	N/A	RNAseq	2048113
223	1	3	18	AA	RNAseq	2048114
224	1	2	19	HAW	RNAseq	2048115
226	4	1	21	AA	RNAseq	2048117
227	7	2	21	W	RNAseq	2048118
228	1	3	19	AA	RNAseq	2048119

AA = African American, Asian = Asian or Asian American,

H = Hispanic or Latino, HAW = Native Hawaiian, W = White, N/A = not available.

As there are few datasets with circular RNAs cataloged in clinically-relevant biofluids, we expect this data to contribute to the characterization of circRNAs in young, healthy males. While this might be a direct comparator for concussions, or other diseases more prevalent in young men, we also expect this dataset to help begin to fill out a broader assessment of circRNAs present in healthy populations.

## Methods

### Sample collection and participants

Samples were collected from healthy, male volunteers, ages 18–25, with consent and approval from the Western Institutional Review Board (WIRB) study ID #1307009395. All participants provided written consent prior to enrollment. We obtained plasma (n = 134) and urine (n = 114) samples from 54 healthy male volunteers. In 71.4% of participants, both biofluid types were collected from the same individual. Blood samples were collected in EDTA tubes, and urine was collected in sterile cups. After collection, samples were placed in a cooler with ice packs and transported from Arizona State University to the Translational Genomics Research Institute, within 2–3 hours of collection. Blood samples were spun down at 1320 x G for 10 minutes at 4 °C, and 1 mL aliquots of plasma were collected in RNase/DNase free microcentrifuge tubes (VWR) and stored at −80 C. Urine samples were spun at 1900 x G for 10 minutes at 4 °C and 15 mL aliquots were collected in 50 mL conical tubes for storage at 80 °C.

### RNA isolation, library preparation, and sequencing

For plasma samples, total RNA was isolated from 1 mL plasma using the mirVana PARIS RNA and Native Protein Purification Kit (Thermo Fisher, Cat. No.: AM1556) as in Burgos *et al*.^42^, treated with the DNA-free DNA Removal Kit (Thermo Fisher, Cat. No.: AM1906), and purified and concentrated with RNA Clean & Concentrator – 5 columns (Zymo Research, Cat. No.: R1016) by following Appendix C in the kit’s protocol. For urine samples, total RNA was isolated from 15 mL urine using Norgen’s Urine Total RNA Purification Maxi Kit (Slurry Format) (Norgen, Cat. No.: 29600), treated with the RNase-Free DNase Set (Qiagen, Cat. No.: 79254), and concentrated with the speed vacuum. The isolated RNA was quantitated with Quant-iT Ribogreen RNA Assay (Thermo Fisher, Cat. No.: R11490). Samples were not ribo-depleted, double-stranded cDNA was synthesized from 10 ng total RNA with the SMARTer Universal Low Input RNA Kit for Sequencing (Clontech, Cat. No.: 634940) using thirteen PCR cycles. The double-stranded cDNA was quantitated with the Qubit dsDNA HS Assay Kit (Thermo Fisher, Cat. No.: Q32854). For each healthy control sample, Illumina-compatible libraries were synthesized from 2 ng double-stranded cDNA with Clontech’s Low Input Library Prep Kit (Clontech, Cat. No.: 634947) using four mandatory PCR cycles plus ten additional cycles. Each library was measured for size via Agilent’s High Sensitivity D1000 Screen Tape and reagents (Agilent, Cat. No.: 5067–5602 & 5067–5585) and measured for concentration via the KAPA SYBR FAST Universal qPCR Kit (Kapa Biosystems, Cat. No.: KK4824). Libraries were then combined into equimolar pools, and each pool was measured for size and concentration. Pools were clustered onto a paired-end flowcell (Illumina, Cat. No.: PE-401–3001) with a 20% v/v PhiX v3 spike-in (Illumina, Cat. No.: FC-110-3001) and sequenced on Illumina’s HiSeq. 2500 with TruSeq v3 chemistry (Illumina, Cat. No.: FC-401-3002). The first and second reads were each 83 bases.

### CircRNA prediction

Samples were demultiplexed and raw fastqs generated using CASAVA (v1.8.2, Illumina). Raw fastqs were trimmed using cutadapt (v1.9) with a quality score cutoff of 30 and a minimum length of 30 bp^43^. For each sample, 6 different algorithms (Table 2) were used to predict circRNA: KNIFE v1.4^44^, find_circ^21^, MapSplice2^45^, CIRCexplorer^46^, CIRI2^47^, and DCC^48^. Indices of the GRCh37/hg19 genome were created using bwa and STAR v2.4.0j using default parameters^49,50^; bowtie and bowtie2 genome indices were downloaded with the KNIFE package^51,52^. Reads were mapped to the genome with the recommended aligner and alignment parameters for each program: STAR v2.4.0j for DCC and CIRCexplorer, bowtie2 v2.2.1 for find_circ and KNIFE, bowtie v0.12.9 for MapSplice2, and bwa v0.7.13 for CIRI2. CircRNA prediction was then completed with the suggested parameters for each program, with the exception of incorporating a minimum 18nt overlap on either side of the junction. CircRNAs were kept for downstream analysis if they 1) had 2 or more junction counts and 2) were identified in at least 5 samples for each respective program.

**Table 2:** CircRNA program characteristics.

Program	Aligner	Version	Paired-End Read Aware	Annotation Aware	Default Junction Overlap	Adjusted Junction Overlap	Reference
KNIFE	bowtie2	1.4	Yes	Yes	13 nt	18 nt	^44^
find_circ	bowtie2	1.0	No	No	18 nt	—	^21^
MapSplice	bowtie	2.1.8	Yes	Yes	10 nt	18 nt	^45^
CIRCexplorer	STAR	1.1.7	No*	Yes	15 nt**	18 nt	^46^
CIRI	bwa	2.0.1	Yes	Yes	19 nt***	—	^47^
DCC	STAR	0.3.2	Yes	Yes	15 nt**	18 nt	^48^

*The latest version of CIRCexplorer now supports paired-end reads.

**CIRCexplorer and DCC use the STAR chimeric junctions output, so the junction overlap for these tools is set by the splice junction parameters during STAR alignment.

***The default minimum seed length (k) for bwa mem is 19 nucleotides.

### Analysis of predicted circRNA

The version of CIRCexplorer used here does not support paired-end data; therefore, circRNA prediction was performed on each pair separately and then combined for analysis. For each program, BED files containing count expression data were created from the output data. CIRCexplorer, KNIFE, and find_circ output files all produce output files with 0-based coordinates while CIRI2, MapSplice, and DCC output files have 1-based coordinates; therefore, all coordinates were converted to a 0-based system for comparison. BED12 GRCh37 RefSeq gene annotation files were obtained from UCSC (http://genome.ucsc.edu/cgi-bin/hgTables), and bedtools v2.26.0 was used to infer genes from reported backsplice junction genome locations^53^. Data were analyzed using the R v3.3.2 statistical package (https://cran.r-project.org). UpSet plots were generated using the UpSetR v1.3.3 package^54^.

### Quantification of circRNA expression

CircRNA count expression data was obtained from each respective bioinformatic program. Junction reads per million (JRPM) were calculated according to the total number of junction reads found in each sample as identified by STAR (both canonical and chimeric); therefore, JRPM = (circRNA count/junction reads) * 1,000,000. The circular-to-linear ratio (CLR) for each circRNA was calculated as described previously^13,27^, by counting the linear spliced reads identified by STAR on the 5′ and 3′ flanks of each circRNA junction, and dividing the back-spliced read count by the flank with the highest count; therefore, CLR = circRNA count/max (5′ linear junction count, 3′ linear junction count). In order to avoid division by zero, if no linearly spliced reads were detected, a pseudo count of 1 was added to the denominator. The number of reads assigned to the transcriptome was calculated using featureCounts (subread v1.5.1) with the Ensembl75 gene annotation^55^. Differential expression analysis was performed using DESeq. 2 v1.14.1^56^, after filtering to select samples which had detected at least 300 circRNA/sample as well as exclusion of circRNA that were expressed in less than 50% of samples.

### DNA isolation and qRT-PCR

After centrifugation of blood samples, DNA was isolated from the buffy coat using the DNeasy Kit (Qiagen, Cat. No.: 69504). Previously isolated RNA from samples matching those used for library prep were selected for cDNA synthesis. cDNA was synthesized with random hexamers using the SuperScript III First-Strand Synthesis System for RT-PCR following manufacturer’s protocols (Invitrogen, Cat. No.: 18080-051) with three nanograms of total RNA as input, and stored at −20 °C. Inward-facing (crossing the back-splice junction) custom primers were designed with Primer3 and LabReady primers (100 µM in IDTE pH 8.0) were ordered from Integrative DNA Technologies with Standard Desalting Purification^57,58^. Real-time qRT-PCR was performed with SYBR Select Master Mix (Thermo Fisher, Cat. No.: 4472919) on the QuantStudio 7 (Applied Biosystems), with 0.2 µM of primer and 0.2 µL of cDNA template or 2 ng of gDNA template per 10 µL reaction. U6 was used as a positive control and no template controls (NTCs) were used as a negative control. All results are expressed as the mean of three independent reactions, with a standard deviation less than 0.5. The ReadqPCR v1.20.0 and NormqPCR v1.20.0 Bioconductor v3.4 packages were used for qRT-PCR data analysis^59^.

## Data Records

Raw FASTQ files for the RNAseq libraries were deposited into dbGap (accession # phs001258.v2.p1) (https://identifiers.org/dbgap:phs001258.v2.p1)^60^. Data (circRNAs identified across all informatic tools and raw cirRNA expression) are also provided in *figshare*: 10.6084/m9.figshare.c.5420832^61^.

## Technical Validation

### CircRNA set size and genomic alignment

The set size (all circRNA in any sample by one tool) ranges from 1,835 to 7,462 and 163 to 1,349 in plasma and urine, respectively (Table 3). 965 and 72 circRNA were detected across all six tools in plasma and urine, respectively (Fig. 2a,c, red bars; Table 4; full list in figshare File 1 and 2^61^). KNIFE predicted the most circRNA per sample in plasma and urine, while MapSplice predicted the fewest (Table 3). Table 5 displays the correlations between all of the tools, CIRCexplorer and DCC had the highest correlation. 85% (61 of the 72) of the circRNAs found in urine were also detected in plasma (Table 4). Figure 2b**(**plasma) and 2d (urine**)** display the number of detected circRNAs and the number that span introns, exons, and UTRs for both plasma and urine. The majority of circRNA identified in plasma and urine contain at least two exons and span an intron; 671 in plasma and 52 in urine; green bars (Fig. 2b, plasma and 2d, urine). A small number of circRNA are transcribed from a single exon (15 in plasma and 2 in urine).

**Table 3:** CircRNA totals detected across six informatic tools in plasma and urine.

	Plasma		Urine	
	total circRNA	mean circRNA/sample	total circRNA	mean circRNA/sample
CIRCexplorer	6,297	909	1,142	119
CIRI2	6,789	1,075	1,205	131
DCC	7,159	1,009	1,287	132
find_circ	2,916	396	438	44
KNIFE	7,462	1,086	1,349	139
MapSplice	1,835	279	163	17

**Table 4:** Number of circRNA detected in plasma and urine by all 6 bioinformatic tools.

	Plasma (n = 134)	Urine (n = 114)	Both Plasma and Urine
Detected in at least 1 sample	965	72	61
Detected in 10% of samples	964	71	60
Detected in 20% of samples	881	61	51
Detected in 30% of samples	675	41	34
Detected in 40% of samples	538	28	24
Detected in 50% of samples	395	16	16
Detected in 60% of samples	273	14	11
Detected in 70% of samples	177	10	10
Detected in 80% of samples	68	4	2
Detected in 90% of samples	15	2	1
Detected in 100% of samples	0	0	0

**Table 5:** Pearson’s correlation of circRNA expression (JRPM) between informatic tools.

Plasma						
	CIRCexplorer	CIRI	DCC	find_circ	KNIFE	MapSplice
CIRCexplorer	1	0.878	0.945	0.838	0.845	0.798
CIRI	0.878	1	0.882	0.836	0.841	0.908
DCC	0.945	0.882	1	0.843	0.887	0.79
find_circ	0.838	0.836	0.843	1	0.82	0.776
KNIFE	0.845	0.841	0.887	0.82	1	0.773
MapSplice	0.798	0.908	0.79	0.776	0.773	1
**Urine**						
	**CIRCexplorer**	**CIRI**	**DCC**	**find_circ**	**KNIFE**	**MapSplice**
CIRCexplorer	1	0.824	0.916	0.74	0.824	0.767
CIRI	0.824	1	0.869	0.738	0.843	0.817
DCC	0.916	0.869	1	0.793	0.889	0.733
find_circ	0.74	0.738	0.793	1	0.801	0.718
KNIFE	0.824	0.843	0.889	0.801	1	0.709
MapSplice	0.767	0.817	0.733	0.718	0.709	1

### Highly expressed, back-spliced junctions were validated by qRT-PCR

**Fig. 3:**
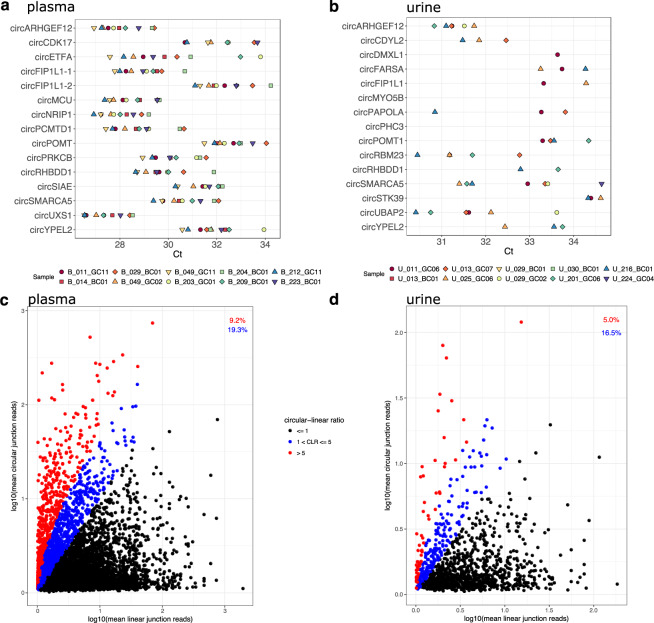
(**a**,**b**) Highly-expressed, predicted back-spliced junctions were validated by qRT-PCR. qRT-PCR validation of the 15 most highly expressed circRNA found in plasma (**a**) and urine (**b**), respectively. Each circRNA was examined in 10 cDNA samples from the same source RNA as sequenced samples. (**c,d**) Circular-linear ratios are higher in plasma than urine. Linear splice junction expression plotted against circular splice junction expression in plasma (**c**) and urine (**d**). Points representing circRNA between 1-fold and 5-fold higher than their linear counterparts are blue; 5x or higher are red.

In order to validate predicted back-spliced junctions by qRT-PCR, we designed inward-facing primers for the 15 most highly expressed circRNA in each biofluid and tested each primer pair in samples from 10 different individuals, using the same source RNA for cDNA synthesis that was used for RNAseq (Fig. 3a,b). Figure 3a shows that the 15 circRNAs are detected in most of the 10 plasma samples. The numbers of samples are described in Table 6, and compared with the RNASeq detection for those circRNAs in the same samples. 13 primer pairs were validated in urine. Detection in urine samples was sparse, with fewer samples positive for each circRNA than for plasma (Fig. 3b and Table 6). For the two back-spliced junctions detected in RNASeq data, but not validated by qRT-PCR in urine (circMYO5B and circPHC3), it is possible that the circRNA primers did not work, or there were qPCR inhibitors in the sample, or the circRNA was not present. Two of the samples did not have enough assigned reads via RNASeq to be included, so the total number of samples was 8. In order to rule out chimeric junctions that might be present in DNA or resemble artifacts introduced during library preparation, we also used genomic DNA (gDNA) from each individual as a negative control. All 15 primer pairs used in the plasma and urine samples were not detected in gDNA (data not shown). Table 7 describes the rank from highest to lowest expression for each of the circRNA validated by qRT-PCR, and compares it with the expression detected with sequencing. Their ranks do not correlate well between the two platforms.

**Table 6:** circRNA detection in 10 samples by qRT-PCR and RNASeq.

qRT-PCR	circRNA Detection	RNASeq	circRNA Detection
plasma	X out of 10 samples tested	plasma	X out of 10 samples tested
**circARHGEF12**	10	**circARHGEF12**	9
**circFIP1L1-1**	10	**circFIP1L1-1**	9
**circMCU**	10	**circMCU**	9
**circRHBDD1**	10	**circRHBDD1**	9
**circSIAE**	9	**circSIAE**	9
**circCDK17**	8	**circCDK17**	9
**circFIP1L1-2**	10	**circFIP1L1-2**	9
**circNRIP1**	10	**circNRIP1**	9
**circPOMT1**	10	**circPOMT1**	9
**circSMARCA5**	10	**circSMARCA5**	9
**circETFA**	10	**circETFA**	7
**circPCMTD1**	10	**circPCMTD1**	6
**circPRKCB**	10	**circPRKCB**	9
**circUXS1**	10	**circUXS1**	9
**circYPEL2**	10	**circYPEL2**	9
**qRT-PCR**	**circRNA Detection**	**qRT-PCR**	**circRNA Detection**
**urine**	**X out of 10 samples tested**	**urine**	**X out of 10 samples tested**
**circPHC3**	0	**circPHC3**	8
**circPOMT1**	4	**circPOMT1**	8
**circRHBDD1**	2	**circRHBDD1**	6
**circSMARCA5**	7	**circSMARCA5**	7
**circYPEL2**	3	**circYPEL2**	7
**circCDYL2**	3	**circCDYL2**	4
**circFARSA**	3	**circFARSA**	7
**circPAPOLA**	3	**circPAPOLA**	7
**circRBM23**	5	**circRBM23**	4
**circUBAP2**	6	**circUBAP2**	6
**circARHGEF12**	6	**circARHGEF12**	7
**circDMXL1**	1	**circDMXL1**	5
**circFIP1L1**	2	**circFIP1L1**	7
**circMYO5B**	0	**circMYO5B**	7
**circSTK39**	3	**circSTK39**	7

**Table 7:** qRT-PCR and RNA-Seq expression of the 15 most highly expressed genes in plasma and urine.

Plasma				
circRNA	mean Ct	qRT-PCR Rank	mean JRPM	RNA-Seq Rank
circUXS1	27.4	1	51.21	15
circNRIP1	27.96	2	89.8	10
circARHGEF12	28.04	3	110.13	5
circMCU	28.53	4	415.78	1
circPCMTD1	28.8	5	60.09	13
circFIP1L1-1	28.99	6	250.67	2
circRHBDD1	29.75	7	217.81	4
circETFA	30.05	8	66.35	11
circPRKCB	30.16	9	65.81	12
circSMARCA5	30.64	10	99.59	9
circSIAE	31.41	11	234.59	3
circYPEL2	31.88	12	51.44	14
circCDK17	32.12	13	102.04	7
circFIP1L1-2	32.36	14	100.46	8
circPOMT1	32.6	15	106.79	6
**Urine**				
**circRNA**	**mean Ct**	**qRT-PCR Rank**	**mean JRPM**	**RNA-Seq Rank**
circARHGEF12	31.27	1	5.27	15
circRBM23	31.46	2	8.21	5
circUBAP2	31.68	3	6.49	10
circCDYL2	31.93	4	6.72	8
circPAPOLA	32.64	5	6.61	9
circSMARCA5	32.72	6	7.85	6
circRHBDD1	33.22	7	10.72	3
circYPEL2	33.25	8	9.3	4
circDMXL1	33.63	9	6.29	11
circPOMT1	33.67	10	28.46	2
circFARSA	33.75	11	7.5	7
circFIP1L1.2	33.8	12	5.61	14
circSTK39	34.45	13	6.24	12
circMYO5B	N/A	14	5.7	13
circPHC3	N/A	15	81.19	1

### Circular-to-linear RNA ratios

While the overall expression of most circRNAs is low compared to their linear counterparts, there are a number of circular RNA transcripts that have been described as more abundant than their linear host, cellularly as well as extracellularly^23,27,62,63^. We examined the circular-to-linear ratio (CLR) of circRNA transcripts found in plasma and urine as described previously; by taking the ratio of the circular, back-spliced junction counts compared to the linear count of the nearest 5′ or 3′ splice junction^13,24,27^. On average, 28.5% of circRNA transcripts in plasma and 21.5% of circRNA transcripts in urine have higher expression than their linear host gene (Fig. 3c, plasma and 3d, urine). Extracellular RNA is often fragmented and may have a 3′ bias^64^. Before examining the expression of circular RNA in relation to their host genes, we calculated the overall 5′ to 3′ coverage of linear transcripts and did not find a bias in our samples.

### Participants sequenced 5 or more times have less inter-sample variation

**Fig. 4:**
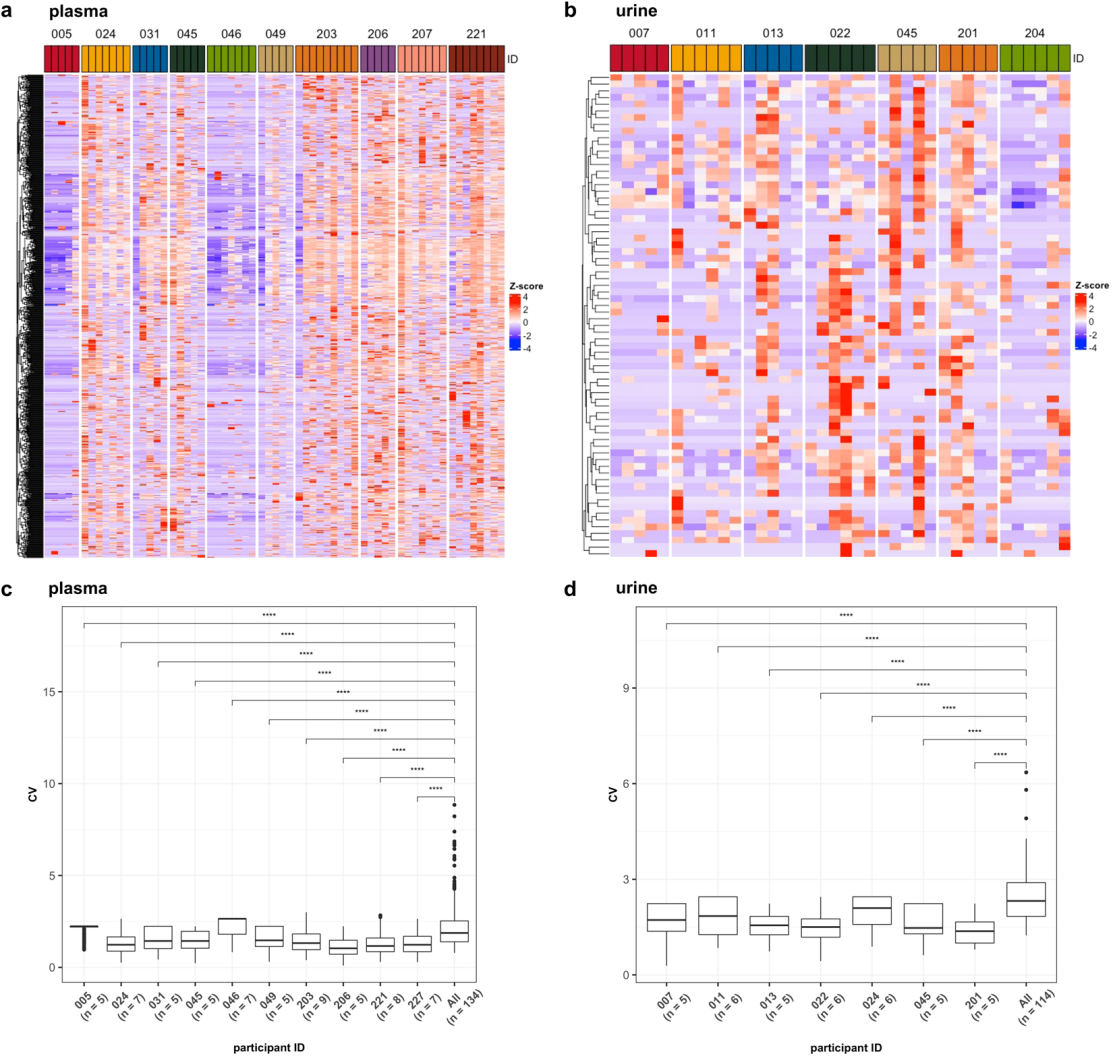
Participants sequenced five or more times have less inter-sample variation. CircRNA populations identified in plasma (**a**,**c**) and urine (**b**,**d**) from participants sampled five or more times. (**a**,**b**) Heatmaps showing the log-normalized JRPM expression of plasma (**a**) and urine (**b**) samples taken longitudinally from the same participant. The coefficient of variation (CV) of circRNA expression is significantly lower across individual participant samples when compared to the entire dataset (**c**, plasma; and **d**, urine). ****p <  = 0.0001.

A notable feature of this dataset is that many participants were sampled longitudinally, allowing for analysis of circRNA stability in individuals versus the entire dataset. Figure 4a,b show longitudinal circRNA expression in the same participants in plasma and urine, respectively. Broadly speaking, the heatmaps demonstrate similar expression patterners in the same participant over time. In order to assess variability within individuals, we calculated the coefficient of variation (CV) of circRNA expression, normalized to junction reads per million (JRPM). Here, we focus on participants sampled on 5 or more occasions over approximately one year. In both plasma and urine, the CV for each individual participant is displayed along with the CV for all participant samples. The data indicate that individuals have a statistically-significant consistency in circRNA expression pattern over time (Fig. 4c, plasma and 4d, urine).

## Usage Notes

As the approach to detecting circRNA from RNA-Seq data differ with available tools, we employed 6 different bioinformatic tools: CIRCexplorer, CIRI2, DCC, KNIFE, find_circ, and MapSplice, in two clinically relevant biofluids, plasma and urine, using 134 and 114 samples, respectively. Most of these circRNA pipelines use an external aligner, such as bowtie, STAR, or bwa, to align reads to the genome and/or transcriptome (Table 2). After alignment, reads that contiguously align to the genome and/or transcriptome are filtered out, and the remaining unmapped reads are further filtered to identify back-spliced junctions. Differences in circRNA identification algorithms include: 1) how paired-end reads and gene annotations are used, if at all, 2) the amount of overlap over the junction that a read must contain, 3) the types of junctions considered, and 4) various filtering steps (Table 1)^65^. We sought to generate a high confidence set of circRNA expressed in plasma and urine with the following requirements for each circRNA: 1) detection in at least 5 samples for each respective biofluid, 2) a minimum 18 nt overlap on either side of the junction, 3) at least two reads spanning the back-spliced junction, and 4) identification by all 6 tested bioinformatic tools as identification can vary widely between tools^66–68^.

We tested alignment parameters and their influence on the detection rate of circRNA and found that the number of input reads, genome mapped reads, and junction reads did not correlate well with the number of circRNA detected per sample; rather the number of reads assigned to the transcriptome had the greatest correlation with the number of circRNA (R^2^ = 0.805; data not shown).

### Supplementary information

Supplemental Data File

## Data Availability

Code used for circRNA identification is available in the Supplemental data. Software versions used for analysis are as follows: STAR v2.4.0j for DCC and CIRCexplorer bowtie2 v2.2.1 for find_circ and KNIFE bowtie v0.12.9 for MapSplice2 bwa v0.7.13 for CIRI2 bedtools v2.26.0 Data were analyzed using the R v3.3.2 statistical package (https://cran.r-project.org). UpSet plots were generated using the UpSetR v1.3.3 package^54^. The ReadqPCR v1.20.0 and NormqPCR v1.20.0 Bioconductor v3.4 packages were used for qRT-PCR data analysis^59^.

## Online-only Table

**Online-only Table 1:** Longituduinal sample collection with circRNAs detected across all informatic tools.

Subject	Collection date	Biofluid	# of circRNA detected	# of circRNA detected in both
1	Time point #1	plasma	119	7
1	Time point #1	urine	39	
2	Time point #1	plasma	348	6
2	Time point #1	urine	33	
4	Time point #1	plasma	220	6
4	Time point #1	urine	20	
4	86 days from Time point #1	plasma	329	10
4	86 days from Time point #1	urine	40	
4	98 days from Time point #1	plasma	104	5
4	98 days from Time point #1	urine	30	
5	Time point #1	plasma	1	1
5	Time point #1	urine	23	
5	65 days from Time point #1	plasma	44	NA
5	72 days from Time point #1	plasma	35	NA
5	98 days from Time point #1	plasma	67	NA
5	121 days from Time point #1	plasma	272	NA
6	Time point #1	plasma	90	6
6	Time point #1	urine	20	
6	51 days from Time point #1	urine	17	NA
6	58 days from Time point #1	plasma	60	NA
6	86 days from Time point #1	plasma	294	6
6	86 days from Time point #1	urine	30	
7	Time point #1	plasma	158	4
7	Time point #1	urine	10	
7	42 days from Time point #1	urine	23	NA
7	58 days from Time point #1	plasma	168	4
7	58 days from Time point #1	urine	20	
7	79 days from Time point #1	urine	12	NA
7*	NA	plasma	79	3
7*	NA	urine	20	
8	Time point #1	plasma	545	NA
8	117 days from Time point #1	plasma	406	NA
10	Time point #1	urine	24	NA
10	7 days from Time point #1	plasma	440	9
10	7 days from Time point #1	urine	45	
10	14 days from Time point #1	plasma	695	NA
10	21 days from Time point #1	plasma	502	NA
11	Time point #1	plasma	38	4
11	Time point #1	urine	44	
11	361 days from Time point #1	plasma	701	NA
11	399 days from Time point #1	plasma	419	NA
11	408 days from Time point #1	urine	8	NA
11	415 days from Time point #1	urine	29	NA
11	436 days from Time point #1	urine	31	NA
11	450 days from Time point #1	urine	38	NA
11	464 days from Time point #1	urine	25	NA
11	485 days from Time point #1	plasma	708	NA
13	Time point #1	plasma	28	2
13	Time point #1	urine	14	
13	408 days from Time point #1	urine	53	NA
13	415 days from Time point #1	urine	58	NA
13	427 days from Time point #1	plasma	829	NA
13	450 days from Time point #1	urine	14	NA
13	457 days from Time point #1	urine	27	NA
14	Time point #1	plasma	208	4
14	Time point #1	urine	7	
14	51 days from Time point #1	urine	23	NA
14	361 days from Time point #1	plasma	577	7
14	361 days from Time point #1	urine	41	
14	436 days from Time point #1	urine	51	NA
14	485 days from Time point #1	plasma	836	NA
15	Time point #1	plasma	543	8
15	Time point #1	urine	52	
19	Time point #1	plasma	199	NA
19	38 days from Time point #1	plasma	474	NA
19	54 days from Time point #1	plasma	556	NA
19	75 days from Time point #1	urine	21	NA
22	Time point #1	urine	12	NA
22	128 days from Time point #1	plasma	57	3
22	128 days from Time point #1	urine	25	
22	361 days from Time point #1	plasma	348	2
22	361 days from Time point #1	urine	21	
22	450 days from Time point #1	urine	58	NA
22	457 days from Time point #1	urine	52	NA
22	464 days from Time point #1	urine	33	NA
23	Time point #1	urine	19	NA
24	Time point #1	plasma	836	NA
24	9 days from Time point #1	plasma	424	5
24	9 days from Time point #1	urine	30	
24	16 days from Time point #1	urine	1	NA
24	28 days from Time point #1	plasma	818	NA
24	51 days from Time point #1	plasma	406	3
24	51 days from Time point #1	urine	11	
24	58 days from Time point #1	plasma	645	6
24	58 days from Time point #1	urine	21	
24	65 days from Time point #1	plasma	508	9
24	65 days from Time point #1	urine	34	
24	97 days from Time point #1	plasma	508	NA
24	Time point #1	urine	23	NA
25	Time point #1	plasma	538	NA
25	54 days from Time point #1	plasma	71	1
25	54 days from Time point #1	urine	5	
25	89 days from Time point #1	urine	20	NA
29	Time point #1	plasma	464	NA
29	47 days from Time point #1	urine	21	NA
29	54 days from Time point #1	plasma	565	NA
29	103 days from Time point #1	urine	11	NA
30	Time point #1	plasma	430	11
30	Time point #1	urine	46	
30	7 days from Time point #1	plasma	777	NA
30	28 days from Time point #1	urine	30	NA
30	49 days from Time point #1	plasma	689	NA
31	Time point #1	plasma	158	7
31	Time point #1	urine	32	
31	114 days from Time point #1	plasma	413	NA
31	121 days from Time point #1	plasma	502	NA
31	361 days from Time point #1	plasma	448	NA
31	399 days from Time point #1	plasma	658	NA
31	464 days from Time point #1	urine	53	NA
36	Time point #1	urine	21	NA
39	Time point #1	plasma	410	8
39	Time point #1	urine	38	
39	51 days from Time point #1	plasma	172	4
39	51 days from Time point #1	urine	28	
39	361 days from Time point #1	plasma	609	NA
39	408 days from Time point #1	urine	26	NA
39	471 days from Time point #1	plasma	611	NA
42	Time point #1	plasma	31	NA
44	Time point #1	urine	21	NA
44	121 days from Time point #1	plasma	327	6
44	121 days from Time point #1	urine	17	
45	Time point #1	plasma	413	10
45	Time point #1	urine	30	
45	107 days from Time point #1	plasma	343	8
45	107 days from Time point #1	urine	19	
45	361 days from Time point #1	plasma	824	11
45	361 days from Time point #1	urine	51	
45	415 days from Time point #1	urine	13	NA
45	427 days from Time point #1	plasma	423	NA
45	436 days from Time point #1	urine	54	NA
45	496 days from Time point #1	plasma	513	NA
46	Time point #1	plasma	42	1
46	Time point #1	urine	31	
46	14 days from Time point #1	plasma	28	NA
46	14 days from Time point #1	urine	12	NA
46	21 days from Time point #1	plasma	117	4
46	21 days from Time point #1	urine	14	
46	28 days from Time point #1	plasma	271	7
46	28 days from Time point #1	urine	41	
46	56 days from Time point #1	plasma	278	NA
46	63 days from Time point #1	plasma	116	NA
46	70 days from Time point #1	plasma	122	NA
48	Time point #1	plasma	27	NA
48	49 days from Time point #1	plasma	135	4
48	49 days from Time point #1	urine	13	
49	Time point #1	plasma	41	NA
49	47 days from Time point #1	plasma	468	5
49	47 days from Time point #1	urine	11	
49	96 days from Time point #1	plasma	560	4
49	96 days from Time point #1	urine	7	
49	103 days from Time point #1	urine	2	NA
49	117 days from Time point #1	plasma	408	NA
49	124 days from Time point #1	plasma	478	NA
170	Time point #1	plasma	183	5
170	Time point #1	urine	22	
201	Time point #1	plasma	688	10
201	Time point #1	urine	42	
201	47 days from Time point #1	urine	57	NA
201	54 days from Time point #1	urine	53	NA
201	89 days from Time point #1	urine	24	NA
201	103 days from Time point #1	plasma	598	9
201	103 days from Time point #1	urine	28	
201	117 days from Time point #1	plasma	489	NA
202	Time point #1	urine	17	NA
202	47 days from Time point #1	urine	11	NA
202	54 days from Time point #1	urine	4	NA
203	Time point #1	plasma	70	NA
203	38 days from Time point #1	plasma	585	NA
203	47 days from Time point #1	plasma	634	9
203	47 days from Time point #1	urine	23	
203	54 days from Time point #1	plasma	361	4
203	54 days from Time point #1	urine	15	
203	66 days from Time point #1	plasma	511	NA
203	89 days from Time point #1	plasma	818	11
203	89 days from Time point #1	urine	13	
203	96 days from Time point #1	plasma	470	6
203	96 days from Time point #1	urine	18	
203	103 days from Time point #1	plasma	488	NA
203	110 days from Time point #1	plasma	812	NA
204	Time point #1	plasma	83	NA
205	Time point #1	plasma	357	4
205	Time point #1	urine	46	
206	Time point #1	plasma	375	NA
206	89 days from Time point #1	plasma	651	NA
206	103 days from Time point #1	plasma	654	NA
206	110 days from Time point #1	plasma	784	NA
206	117 days from Time point #1	plasma	717	NA
207	Time point #1	plasma	786	NA
207	47 days from Time point #1	urine	13	NA
207	54 days from Time point #1	plasma	814	NA
208	Time point #1	plasma	647	NA
209	Time point #1	plasma	586	NA
209	103 days from Time point #1	urine	52	NA
210	Time point #1	plasma	667	NA
210	47 days from Time point #1	urine	26	NA
211	Time point #1	plasma	398	NA
212	Time point #1	plasma	463	NA
213	Time point #1	urine	60	NA
213	7 days from Time point #1	urine	37	NA
213	19 days from Time point #1	plasma	431	NA
213	28 days from Time point #1	urine	50	NA
214	Time point #1	urine	9	NA
214	103 days from Time point #1	urine	31	NA
215	Time point #1	urine	15	NA
216	Time point #1	plasma	509	8
216	Time point #1	urine	35	
216	89 days from Time point #1	urine	28	NA
216	96 days from Time point #1	urine	30	NA
218	Time point #1	urine	18	NA
218	117 days from Time point #1	plasma	393	NA
220	Time point #1	plasma	707	NA
221	Time point #1	plasma	377	NA
221	9 days from Time point #1	plasma	511	6
221	9 days from Time point #1	urine	27	
221	16 days from Time point #1	urine	63	NA
221	28 days from Time point #1	plasma	365	NA
221	51 days from Time point #1	plasma	838	NA
221	58 days from Time point #1	plasma	773	NA
221	65 days from Time point #1	urine	13	NA
221	72 days from Time point #1	plasma	587	NA
221	79 days from Time point #1	plasma	760	NA
221	86 days from Time point #1	plasma	745	NA
222	Time point #1	plasma	292	NA
223	Time point #1	urine	7	NA
223	89 days from Time point #1	urine	41	NA
223	103 days from Time point #1	plasma	588	4
223	103 days from Time point #1	urine	28	
224	Time point #1	plasma	774	NA
224	66 days from Time point #1	urine	51	NA
224	103 days from Time point #1	urine	22	NA
226	Time point #1	plasma	413	NA
226	38 days from Time point #1	plasma	791	NA
226	54 days from Time point #1	urine	8	NA
226	103 days from Time point #1	plasma	435	NA
226	124 days from Time point #1	plasma	568	NA
227	Time point #1	plasma	813	NA
227	38 days from Time point #1	plasma	673	NA
227	54 days from Time point #1	plasma	491	9
227	54 days from Time point #1	urine	21	
227	66 days from Time point #1	plasma	666	NA
227	89 days from Time point #1	plasma	445	NA
227	96 days from Time point #1	urine	54	NA
227	103 days from Time point #1	plasma	619	NA
227	135 days from Time point #1	plasma	570	NA
228	Time point #1	plasma	774	11
228	Time point #1	urine	44	
228	35 days from Time point #1	urine	52	NA
228	42 days from Time point #1	urine	54	NA

*denotes samples collected on an unspecified date
