# Supplementary material for: Extracellular circular RNA profiles in plasma and urine of healthy, male college athletes
Source: Sci Data. 2021 Oct 28;8:276. doi: 10.1038/s41597-021-01056-w (PMC8553830; doi:10.1038/s41597-021-01056-w)
Supplement: Supplementary file 1 — Supplemental Data File [file 41597_2021_1056_MOESM1_ESM.docx]

# circRNA tool parameters

## STAR v2.4.0j

STAR \

--runThreadN ${P} \

--genomeDir human_GRCh37/82bp_overhang \

--outSAMtype BAM SortedByCoordinate \

--readFilesIn ${R1} ${R2} \

--outFileNamePrefix ${PREFIX} \

--outReadsUnmapped Fastx \

--outSJfilterOverhangMin 18 18 18 18 \

--alignSJoverhangMin 18 \

--alignSJDBoverhangMin 18 \

--outFilterMultimapNmax 20 \

--outFilterScoreMin 1 \

--outFilterMatchNmin 1 \

--outFilterMismatchNmax 2 \

--seedSearchStartLmax 30 \

--chimSegmentMin 18 \

--chimScoreMin 15 \

--chimScoreSeparation 10 \

--chimJunctionOverhangMin 18 \

--outFilterType BySJout \

--genomeLoad NoSharedMemory

## KNIFE v1.4

#### -- dependencies --

python v2.7.3

R v3.0.3

samtools v1.2

bowtie v0.12.9

#### -- parameters --

READ_STYLE=complete

OVERLAP=18

MODE=bam_sort

NTRIM=55

#### -- running KNIFE --

completeRun.sh ${READ_DIR} ${READ_STYLE} ${ALIGN_PARDIR} ${DATASET_NAME} ${OVERLAP} ${MODE} ${REPORTDIR_NAME} ${NTRIM} ${BT1_INDEX} ${BT2_INDEX} ${SCRIPT_DIR}

## find_circ v1.0

#### -- dependencies --

python v2.7.3

bowtie2 v2.1.0

samtools v1.2

#### --- map against genome, sort ---

bowtie2 -p 8 --very-sensitive --mm --score-min=C,-15,0 -x ${GENOME_INDEX} -q -1 ${R1} -2 ${R2} 2> bowtie2.log > ${SAMPLE_NAME}.sam

samtools view -hbu ${SAMPLE_NAME}.sam | samtools sort -o - ${SAMPLE_NAME} > ${SAMPLE_NAME}.bam

#### --- keep unmapped, split good quality reads into anchors for independent mapping ---

samtools view -hf 4 ${SAMPLE_NAME}.bam | samtools view -b - > unmapped_${SAMPLE_NAME}.bam

unmapped2anchors.py unmapped_${SAMPLE_NAME}.bam | gzip > anchors.gfa.gz

#### --- screen for spliced reads, either linear or head-to-tail (circular) splicing ---

zcat anchors.gfa.gz | bowtie2 -p 8 --reorder --mm --score-min=C,-15,0 -q -x bt2/hg19_genome -U - | find_circ.py -G UCSC_genome -p splice -s sites.log > sites.bed 2> sites.reads

grep circ sites.bed | grep -v chrM | sum.py -2,3 | scorethresh.py -16 1 | scorethresh.py -15 2 | scorethresh.py -14 0 | scorethresh.py 7 2 | scorethresh.py 8 35 | scorethresh.py 9 35 | scorethresh.py -17 100000 > circ_candidates.bed

## MapSplice v2.1.8

#### -- dependencies --

python v2.7.3

#### -- running MapSplice --

python mapsplice.py -p 15 -o ${OUTPUT_DIRECTORY} -x built_bowtie_index --bam --fusion --min-fusion-distance 200 --gene-gtf gencode.v16.annotation.gtf -c UCSC_genome -1 ${R1} ${R2}

## CIRCexplorer v1.1.7

#### -- dependencies --

python v2.7.10

#### -- running CIRCexplorer --

star_parse.py Chimeric.out.junction fusion_junction.txt

CIRCexplorer.py -j fusion_junction.txt -o ${PREFIX} -g Homo_sapiens.GRCh37.75.dna.toplevel.fa -A hgTableUCSC.txt

## CIRI v2.0.1

#### -- dependencies --

perl v5.14.2

samtools v1.3.1

bwa v0.7.13

#### -- running CIRI --

bwa mem -t 16 -T 19 Homo_sapiens.GRCh37.75.dna.toplevel ${R1} ${R2} > ${SAMPLE_NAME}.sam

perl CIRI_v2.0.1.pl -I ${SAMPLE_NAME}.sam -O ${SAMPLE_NAME}.ciri -F Homo_sapiens.GRCh37.75.dna.toplevel.fa -A Homo_sapiens.GRCh37.75.gtf

## DCC v0.3.2

#### -- dependencies --

python v2.7.3

#### -- running DCC --

python DCC/main.py @data/chimeric_junctions -mt1 @data/chimeric_junctions_mate1 -mt2 @data/chimeric_junctions_mate2 -D -N -R hg19_combined_repeats.gtf -an Homo_sapiens.GRCh37.75.gtf -Pi -F -M -Nr 2 1 -fg -G -A Homo_sapiens.GRCh37.75.dna.toplevel.fa
